# Development and Validation of a Docking-Based Virtual Screening Platform for the Identification of New Lactate Dehydrogenase Inhibitors

**DOI:** 10.3390/molecules20058772

**Published:** 2015-05-15

**Authors:** Carlotta Granchi, Alice Capecchi, Gianluca Del Frate, Adriano Martinelli, Marco Macchia, Filippo Minutolo, Tiziano Tuccinardi

**Affiliations:** Department of Pharmacy, University of Pisa, 56126 Pisa, Italy; E-Mails: carlotta.granchi@farm.unipi.it (C.G.); alice.capecchi@outlook.it (A.C.); gianluca.delfrate@sns.it (G.D.F.); adriano.martinelli@farm.unipi.it (A.M.); marco.macchia@farm.unipi.it (M.M.); filippo.minutolo@farm.unipi.it (F.M.)

**Keywords:** LDH inhibitors, virtual screening, docking

## Abstract

The human muscle isoform of lactate dehydrogenase (*h*LDH5) is one of the key enzymes of the glycolytic process. It is overexpressed in metastatic cancer cells and is linked to the vitality of tumors in hypoxic conditions. With the aim of identifying new *h*LDH5 inhibitors, a fully automated docking-based virtual screening platform was developed by considering different protein conformations and the consensus docking strategy. In order to verify the reliability of the reported platform, a small database of about 10,000 compounds was filtered by using this method, and the top-ranked compounds were tested for their *h*LDH5 inhibition activity. Enzymatic assays revealed that, among the ten selected compounds, two proved to efficiently inhibit enzyme activity with IC_50_ values in the micromolar range. These results demonstrate the validity of the methodologies we followed, encouraging the application of larger virtual screening studies and further refinements of the platform. Furthermore, the two active compounds herein described may be considered as interesting leads for the development of new and more efficient LDH inhibitors.

## 1. Introduction

**Figure 1:**
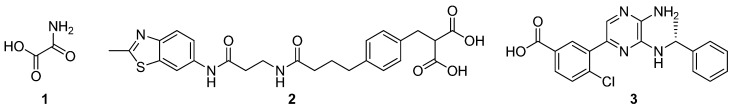
Representative LDH inhibitors deposited in the PDB in complex with *h*LDH5.

Human lactate dehydrogenase (*h*LDH) catalyzes the reduction of pyruvate to lactate (and the reverse reaction) in the presence of a cofactor (NADH or NAD^+^). This enzyme may exist as five functional tetrameric isoforms (*h*LDH1-5), composed of the various combinations of the two monomeric subunits: LDH-A and LDH-B. Over the past few years, this enzyme has been increasingly considered as a potential target for therapeutic agents, including antimalarial and antitumor agents [1]. In particular, *h*LDH5 (LDH-A_4_) catalyzes a crucial step in the glycolytic pathway, which is found to be greatly enhanced in many invasive tumors and, therefore, may determine a sufficient therapeutic window for perspective therapeutic agents interfering with the peculiar metabolism of cancer cells [2,3]. As a matter of fact, numerous examples of *h*LDH5 inhibitors have been recently reported [4]. Our group discovered a new class of *N*-hydroxyindole-based inhibitors of *h*LDH5 [5,6,7], which were demonstrated to specifically interact with this protein by using a self-referencing external cavity laser biosensor technology [8]. Then, we have further functionalized these compounds with sugar portions in order to enhance the uptake by cancer cells, thus exploiting a dual targeting of the Warburg effect [9,10], by following a strategy that was successfully exploited in many other types of antitumor agents [11]. Among the various virtual screening (VS) strategies, docking-based VS is one of the most widely-applied approaches [12]. In the Protein Data Bank [13], some *h*LDH5 X-ray crystal structures have been deposited. One of the first published structures described the interaction of oxamate (**1**, Figure 1) in the pyruvate binding site [14]; furthermore, other structures have been recently reported, such as the complex of *h*LDH5 with a malonic derivative [15] (**2**, Figure 1) and with (*R*)-3-(5-amino-6-((1-phenylethyl)amino)pyrazin-2-yl)-4-chlorobenzoic acid [16] (**3**, Figure 1). Given the presence of these deposited structures, a docking-based VS strategy could be profitably applied. 

Usually, molecular docking can be defined as an optimization task to identify the ligand conformation bound to the target with the most favorable binding energy. However, this is a challenging task mainly due to ligand and protein flexibilities. With regards to the protein flexibility, two different conformational states of *h*LDH5 have been reported, a closed and an open conformation [1]; furthermore, different ligands are able to interact in the presence and/or absence of the NADH co-factor. On these bases, an ensemble VS docking strategy could be a possible way for identifying new *h*LDH5 inhibitors. Very recently, we reported an evaluation study of the consensus docking approach [17]. By using this kind of approach, one ligand is docked into the target protein by means of different docking procedures. Then, among the different best-ranked poses (originated by the different docking procedures) the pose in common with the largest number of docking procedures is considered as the best docking pose. From a qualitative point of view, previous results highlighted that consensus docking was able to predict the ligand binding pose better than the single docking evaluation [17]. Furthermore, concerning the VS studies, the results suggested that this approach performed as well as the best available methods found in the literature, and it was also able to experimentally identify new active molecules. Taking together all of these data, in the present study, we report the development of a VS platform based on a mixed ensemble/consensus docking approach.

## 2. Results and Discussion

An analysis of the various deposited X-ray crystal structures of *h*LDH5 complexed with ligands clearly support the hypothesis that there are different binding methods for inhibiting this enzyme. In 2001, Read and co-workers reported the complex between *h*LDH5 and **1** [14]; in this structure, the ligand strongly interacted with R169; the enzyme showed a closed conformation, and NADH was placed in the co-factor binding site. In 2012 Ward and co-workers reported a malonic derivative (**2**), which interacted with the closed conformation of *h*LDH5 by displacing both the substrate and the NADH co-factor [15]. Finally, in 2013, Fauber and co-workers reported a 2-amino-5-aryl-pyrazine derivative (**3**), which interacted with an open conformation of *h*LDH5 and also showed important interactions with the NADH co-factor; in fact, in this case, the co-factor was still present in the crystal structure [16]. On the basis of this analysis, it was possible to assess that *h*LDH5 inhibitors could interact with both the closed and the open conformation of the enzyme, either in the presence or in the absence of the NADH co-factor. Following this hypothesis, four different protein structures were built for our docking calculations, so that all of the combinations of the open/closed conformation and of the NADH presence/absence were considered. As mentioned above, we have recently reported a consensus-docking reliability analysis by using ten docking procedures [17]. However, the main Achilles’ heel of the consensus docking approach is the long computing time required, so that, unfortunately, large libraries of compounds require a large amount of CPU time. One of the possible solutions to this problem could be the reduction of the number of applied docking procedures. Consequently, in this study, we applied four different docking procedures that corresponded to the application of four different docking software. In order to validate this approach, an enriched VS analysis was carried out. To our knowledge, no enriched database consisting of LDH inhibitors and decoys is presently available in the literature. To build up this database, small molecules for which an LDH inhibition assay was reported in the literature were analyzed. About 200 compounds were identified as LDH inhibitors able to interact with the open conformation of the enzyme in the presence of NADH, and 93 compounds showing an IC_50_ lower than 15 µM were selected as active LDH inhibitors [16,18,19,20,21]. As regards the choice of decoys, we downloaded all of the decoys included in the Maximum Unbiased Validation datasets reported by Rohrer and Baumann [22]. A total of 10,000 molecules were randomly selected from the compounds with a molecular weight between 315 and 560 g/mol that corresponded to the molecular weight possessed by the active molecules included in our dataset. The so-obtained enriched database consisted of 93 active molecules (see Table S1) and 10,000 decoys and was then used to assess the ability of the consensus docking procedure in separating LDH inhibitors from decoys. The performance evaluation was carried out using well-established accepted metrics, such as the enrichment factor (EF) and the AUC of the ROC curve. The 10,093 molecules were thus docked into the LDH open conformation in the presence of NADH by using the four docking procedures and analyzing the consensus docking results. As shown in Table 1, a consensus docking level of four resulted in an EF of 40.7, which corresponded to 37.5% of the maximum reachable EF value. With regards to the AUC, it showed a value of 0.84. Focusing the attention on the active molecules, only 12 out of 93 showed a consensus of four. These results confirmed that the consensus docking protocol has the disadvantage of generating false negatives.

**Table 1:** Consensus docking results for the enriched database analysis. EF, enrichment factor.

Consensus Level	Actives	Decoys	EF
**1**	93	10,000	1.0
**2**	75	1952	3.7
**3**	43	177	19.5
**4**	12	20	40.7
**AUC**	0.84		

**Figure 2:**
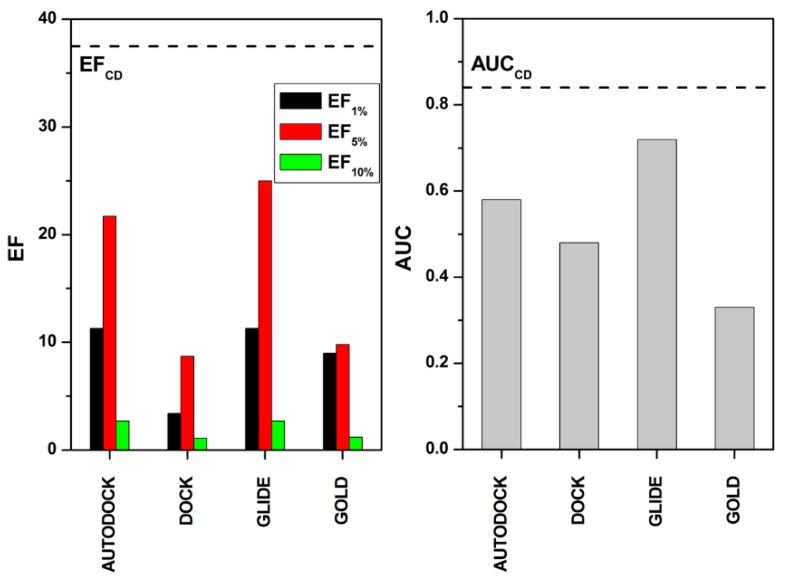
AUC (right plot) and EF (left plot) analysis for the four docking procedures. The black line indicates the EF and AUC values obtained by applying the consensus docking strategy.

The consensus docking results were then compared to those obtained by considering each of the four docking procedures as an independent evaluation and calculating the AUC and EF values generated by the docking-based scoring results. As shown in Figure 2, all of the EF and AUC values obtained for each docking procedure were worse than that obtained for the consensus level of four, thus suggesting that none of the docking procedures were able to filter the enriched database as efficiently as the consensus docking approach.

**Figure 3:**
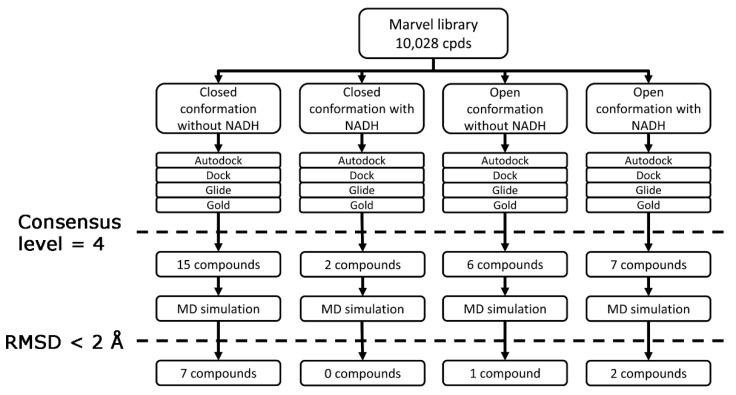
Schematic diagram of the virtual screening workflow.

In order to provide a preliminary experimental VS reliability test, the University of Illinois Marvel library, a collection of about 10,000 compounds, seemed to be an optimal and accessible set of compounds to test our procedure, so it was filtered by using this platform (Figure 3). A library of 10,000 molecules can be considered very small with today’s computing power. However, this VS study was a preliminary analysis, and furthermore, due to the calculation requirements of the consensus docking procedure, the analysis of this library required a total of about 160,000 docking calculations. The Marvel compounds were thus docked into the four protein structures by using the four different docking procedures, and the compounds possessing a consensus level of four for at least one protein structure were further taken into account. The four protein structures were treated as independent of each other. In this way, compounds that showed a consensus level of four into at least one of the four protein structures and that bound differently in the four proteins forms were also selected.

**Figure 4:**
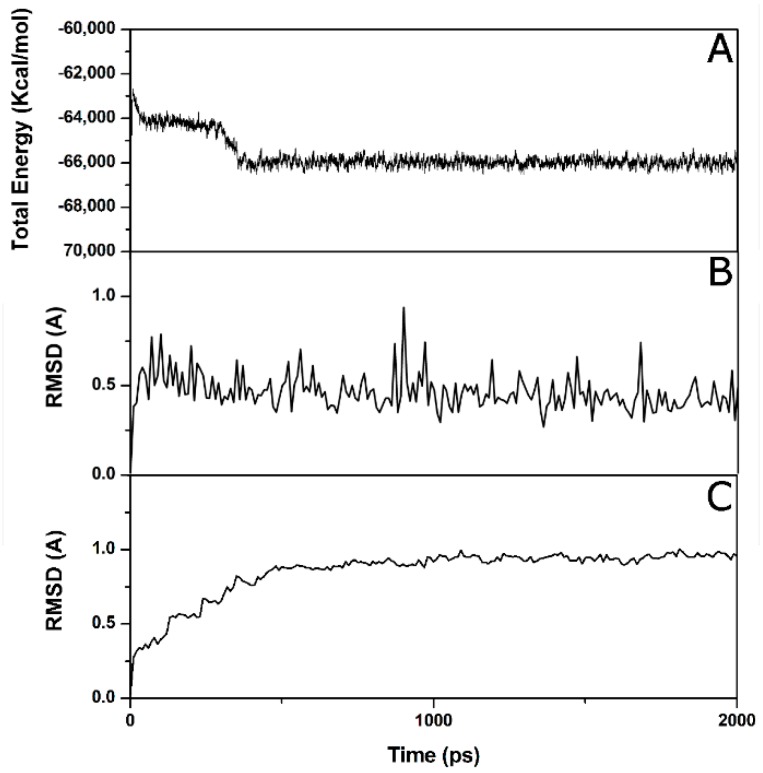
Analysis of the MD simulation of Compound **1** complexed with *h*LDH5: (**A**) total energy of the system *vs.* time; (**B**) RMSD of the ligand from the starting model structure during the simulation; (**C**) RMSD of the heavy atoms of the protein from the starting model structure during the simulation.

The 30 compounds that showed a consensus level of four were subjected to an MD simulation with the aim of verifying the stability of the docking pose. To set up the MD simulation protocol, the complex between *h*LDH5 and **2** (4AJP [15] PDB code) was used as a test. The ligand-protein complex was subjected to a total of 2 ns of MD simulation; as shown in Figure 4, after about 500 ps, the system reached an equilibrium, since the total energy for the last 1.5 ns remained approximately constant. By analyzing the RMSD of the ligand’s position with respect to the starting structures during the simulation, we observed an average RMSD of 0.5 Å, whereas with regards to the analysis of the heavy atoms of the protein, in agreement with the energy analysis after about 500 ps, it showed a stable RMSD value of about 0.9 Å.

The 30 compounds obtained by the previous VS steps were subjected to MD simulation using the protocol described above, and all of the ligands that showed an average RMSD value lower than 2 Å were further considered. The application of an MD time length of 2 ns and the usage of a 2-Å average RMSD threshold was selected, because these two parameters were profitably used in another consensus docking VS study [23]. About 70% of the compounds were rejected by using this filter, and the remaining ten compounds were collected at the University of Illinois in order to submit them to experimental enzyme inhibition assays. Unfortunately, Compounds **VS1**, **VS2** and **VS3** of Table 2 were no longer available; therefore, they were synthesized in our lab.

**Scheme 1:**
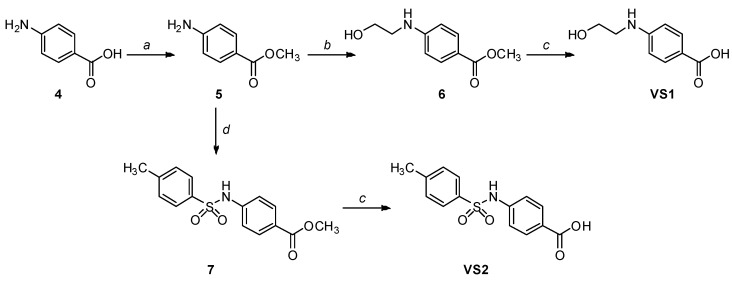
Synthesis of compounds **VS1** and **VS2**.

The synthesis of the first two compounds, **VS1** and **VS2** (Scheme 1), started from commercially available *p*-aminobenzoic acid **4**, which was subjected to a classical Fischer esterification in methanol with catalytic sulfuric acid to protect the carboxylic acid group for the next reaction steps. The resulting methyl ester **5** was then reacted with neat 2-iodoethanol to alkylate the aniline group with a 2-hydroxyethyl chain. Finally, the methyl ester group of Compound **6** was hydrolyzed under basic aqueous conditions to give the desired Compound **VS1**. Intermediate **5** was also condensed with *p*-toluenesulfonyl chloride in the presence of 4-(dimethylamino)pyridine (DMAP) and pyridine to produce sulfonamide **7**, whose saponification yielded Compound **VS2**.

*Reagents and conditions:* (**a**) MeOH, concentrated H_2_SO_4_, reflux, 48 h; (**b**) 2-iodoethanol, neat, 90 °C, 6 h; (**c**) aq. LiOH 2 N, THF/MeOH 1:1, RT, 48 h; (**d**) *p*-toluenesulfonyl chloride, pyridine, catalytic 4-(dimethylamino)pyridine (DMAP), CH_2_Cl_2_, 0 °C to RT, 17 h.

**Scheme 2:**
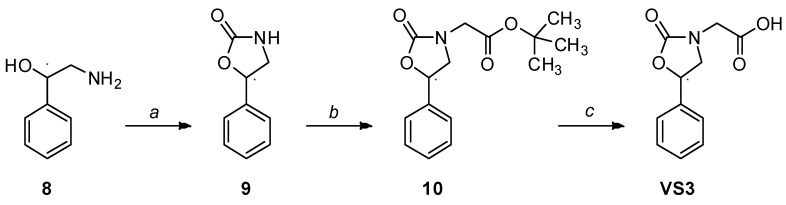
Synthesis of compound **VS3**.

An intramolecular cyclization of commercially available 2-amino-1-phenylethanol **8** in the presence of *N*,*N*-carbonyldiimidazole (CDI) and imidazole gave the oxazolinone **9** (Scheme 2), which was then *N*-alkylated with sodium hydride and *tert*-butyl bromoacetate. Standard deprotection of the *tert*-butyl ester of Compound **10** with trifluoroacetic acid afforded the desired Compound **VS3**.

*Reagents and conditions*: **a**, *N*,*N*-carbonyldiimidazole (CDI), imidazole, CH_2_Cl_2_, RT, 20 h; **b**, NaH, *tert*-butyl bromoacetate, dry DMF, RT, 4 h; **c**, CF_3_COOH, CH_2_Cl_2_, RT, 26 h.

The ten compounds were then subjected to *h*LDH5 inhibition assays together with reference inhibitor galloflavin, oxamic acid [24], *N*-hydroxyindole-based compounds NHI-1 and NHI-2 [25], which were used as positive controls. As shown in Table 2, two out of the ten tested compounds showed appreciable LDH inhibitory activities (**VS6** and **VS8**), with IC_50_ values of about 250 µM. It is worth noting that Compound **VS6** was derived from the closed LDH conformation without the NADH co-factor, whereas Compound **VS8** was derived from the open LDH conformation in the presence of NADH. The docking scores obtained for the ten compounds were also used to rank them; however, none of the four scoring results were able to correctly rank-order the ten final compounds.

**Table 2:** Structure and activity of the tested compounds.

	Structure	*h*LDH5, IC_50_ (µM)
**VS1**	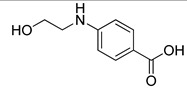	>500
**VS2**	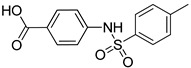	>500
**VS3**	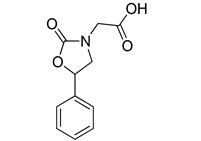	>500
**VS4**	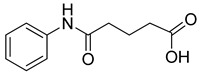	>500
**VS5**	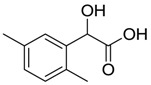	>500
**VS6**	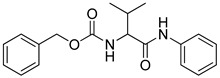	245.7 ± 10.8
**VS7**	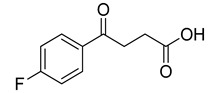	>500
**VS8**	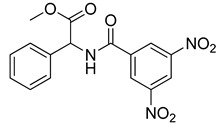	268.6 ± 58.1
**VS9**	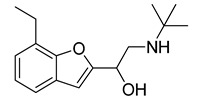	>500
**VS10**	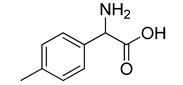	>500
**galloflavin**	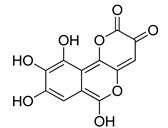	91.7 ± 10.7
**oxamic acid**	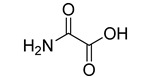	97.7 ± 11.2
**NHI-1**	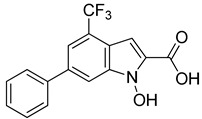	51.7 ± 4.2
**NHI-2**	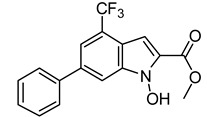	10.8 ± 3.5

**Figure 5:**
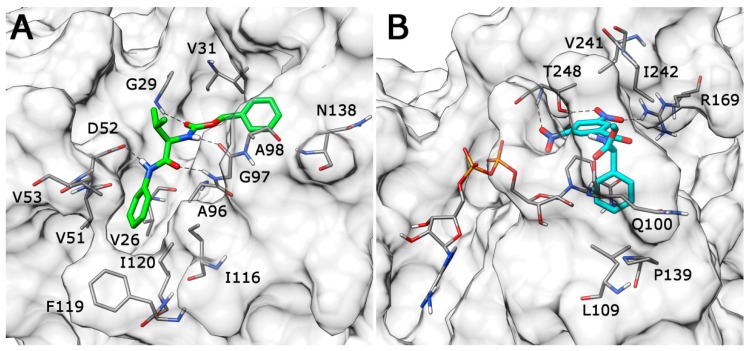
Minimized average structures of Compounds **VS6** (**A**) and **VS8** (**B**) docked into *h*LDH5.

As shown in Figure 5A, Compound **VS6** occupied the NADH binding site; the *N*-phenylacetamide portion of the molecule showed two H-bonds with D52 and G97 and lipophilic interactions with V26, V51, V53, A96, I116, F119 and I120. The isopropyl group was exposed to the solvent and did not show important interactions, whereas the benzyl carbamate portion of the molecule showed a lipophilic interaction with V31 and two H-bonds with the backbone of G29 and G97. Differently from **VS6**, Compound **VS8** occupied the substrate binding region of the enzyme (Figure 5B); the 3,5 dinitrobenzene portion of the molecule showed three H-bonds with R169 and T248 and lipophilic interactions with V241. The amide portion did not show important interactions, whereas the methyl-2-phenylacetate fragment showed one H-bond with the nitrogen backbone of Q100 and lipophilic interactions with L109 and P139.

Among these two newly-identified LDH inhibitors, **VS8** appeared to be the most promising compound, because it occupies the substrate binding region and interacts with key residues, such as T248 and R169. In order to suggest possible modifications to the **VS8** structure for optimizing its activity, this compound was subjected to fragmental scanning by using the BROOD software [26]. This software is capable of identifying bioisosteric replacements for a query fragment, but it can also identify fragments with progressively less similarity to the original molecular fragment. The 3,5-dinitrobenzene portion of the molecule, which was shown to effectively interact with the substrate binding site of the enzyme, was thus subjected to a fragmental scanning. Starting from a database of about one million fragments, those that showed a possible interaction into the protein binding cavity (see the Materials and Methods Section for details) were considered, and the corresponding molecules were processed with consensus docking/MD screening analysis. Table 3 shows the filtered compounds obtained by using this procedure.

**Table 3:** Potential new active LDH inhibitors. 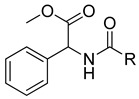

R		
VS8a	VS8b	VS8c
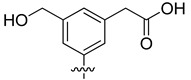	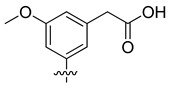	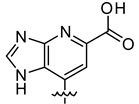

**Figure 6:**
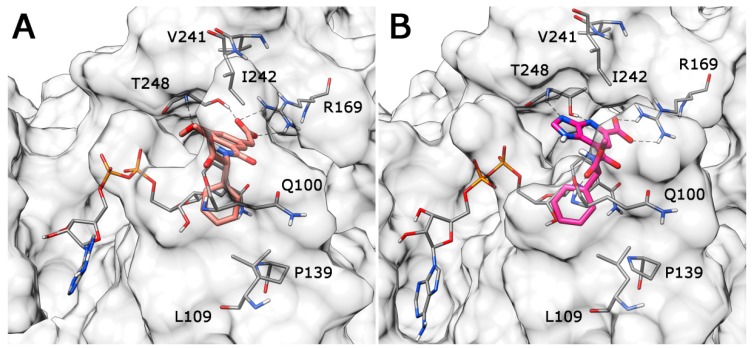
Minimized average structures of compounds **VS8b** (**A**) and **VS8c** (**B**) docked into *h*LDH5.

The analysis highlighted that the substitution of one of the two nitro groups with a carboxylic (**VS8c**) or an acetic substituent (**VS8a** and **VS8b**) could improve the activity. The acetic portion of compounds **VS8a** and **VS8b** showed a strong ionic interaction with R169 and a secondary H-bond with T248, whereas the hydroxyl group of **VS8a** and the methoxy substituent of **VS8b** maintained the H-bond interaction with the nitrogen backbone of T248 (Figure 6A). With regards to the rest of their structures, these molecules showed a disposition very similar to that observed for **VS8**. A similar analysis could also be done for Compound **VS8c**; as shown in Figure 6B, the carboxylic substituent showed a strong ionic interaction with R169; the imidazopyridine nucleus maintained the two H-bonds with the hydroxyl and the nitrogen backbone of T248, whereas the rest of the molecule showed a disposition very similar to that observed for **VS8**.

## 3. Experimental Section

### 3.1. Molecular Modeling

Input generation: In the Protein Data Bank [13] are deposited X-ray crystal structures of *h*LDH5 in the open and closed conformation. In particular, the structure of *h*LDH5 complexed with Compound **2** (4AJP [15] PDB code) corresponded to a closed conformation of the enzyme and did not show NADH co-factor; the *h*LDH5 complexed with **1** (1I10 [14] PDB code) showed a closed conformation and the presence of NADH, whereas the *h*LDH5 complexed with **3** (4M49 [16] PDB code) showed an open conformation of the enzyme and was crystallized in the presence of NADH. For these reasons, for the closed *h*LDH5 conformation in the absence and presence of NADH, 4AJP and 1I10 were used, respectively, and for the open *h*LDH5 conformation in the presence of NADH, the 4M49 structure was used. As there were no deposited structures of the open *h*LDH5 conformation in the absence of NADH co-factor, we generated this structure removing the NADH molecule from the 4M49 structure. For the protein input structures containing NADH, the substrate binding region corresponded to the docking binding site, whereas for the two protein input structures that did not show NADH, the docking binding site corresponded to the fusion of the substrate and co-factor binding site. The Marvel library, a unique collection of over 10,000 compounds stored in the Department of Chemistry at University of Illinois, Urbana-Champaign, was processed by means of the LigPrep software [27], which performs a series of steps that perform conversions, apply corrections to the structures, eliminate unwanted structures and optimize the structures. The so-obtained database was then subjected to the consensus docking calculations into the four *h*LDH5 structures.

Docking procedures: For all docking analyses, only the best-scored pose was taken into account.

AUTODOCK 4.2.3: AUTODOCK Tools utilities [28] were used in order to identify the torsion angles in the ligands, to add the solvent model and assign the Gasteiger atomic charges to proteins and ligands. The regions of interest used by AUTODOCK [29] were defined by considering the reference ligand as the central group of a grid box of 10 Å in the x, y and z directions. A grid spacing of 0.375 Å and a distance-dependent function of the dielectric constant were used for the energetic map calculations. By using the Lamarckian genetic algorithm, the docked compounds were subjected to 20 runs of the AUTODOCK search using 2,500,000 steps of energy evaluation and the default values of the other parameters. 

DOCK 6.5: The molecular surface of the binding site was calculated by means of the MS program [30], generating the Connolly surface with a probe with a radius of 1.4 Å. The points of the surface and the vectors normal to it were used by the Sphgen program in order to build a set of spheres, with radii varying from 1.4–4 Å that describe, from a stereoelectronic point of view, the negative image of the site. Spheres within a radius of 10 Å from the reference ligand were used to represent the site. For each ligand, DOCK 6.5 calculated 500 orientations; among them, the best grid scored was taken into consideration. The grid-based score is based on the non-bonded terms of the molecular mechanic force field.

GLIDE 5.0: The binding site was defined by a rectangular box of 10 Å in the x, y and z directions centered on the ligand. The possibility of imposing a maximum number of atoms a ligand may have if it were to be docked was deactivated, so that all of the ligands were docked independently from the number of their atoms, whereas the GLIDE [31] defaults were used for all other parameters. The docking analysis was carried out using the standard precision (SP) methods.

GOLD 5.1: The region of interest for the docking studies was defined in such a manner that it contained all residues that stayed within 10 Å from the ligand in the X-ray structures; the “allow early termination” command was deactivated, while the possibility for the ligand to flip ring corners was activated. For all other parameters, GOLD [32] defaults were used, and the ligands were subjected to 30 genetic algorithm runs by applying the ChemPLP fitness functions.

Consensus docking evaluation: By applying the four docking software, four different binding dispositions (best-scored docking pose) resulted from the docking of each ligand into each protein binding site. The RMSD of each of these docking poses against the remaining three was evaluated by using the rms_analysis software of the GOLD suite. On this basis, for each ligand docked into each protein binding site, a 4 × 4 matrix was generated reporting the RMSD results. By using an in-house program, these results were clustered, so that among the four results, all of the similar docking poses were clustered together. As a clustering algorithm, we used the complete-linkage method, which is an agglomerative type of hierarchical clustering. This method starts considering each element in a cluster of its own. The clusters are then sequentially combined into larger ones, until all elements are in the same cluster. At each step, the two clusters separated by the shortest distance are combined. We selected an RMSD clustering threshold of 2.0 Å, therefore, the so-obtained clusters contained the group of poses that are less than 2.0 Å away from all others poses belonging to the same cluster. All of the ligands showing a consensus level of four were taken into account.

Database generation: The 18 Maximum Unbiased Validation datasets of decoys were prefiltered by selecting only compounds with a molecular weight between 315 and 560 g/mol. The retained compounds belonging to the different datasets were collected in a unique decoy dataset of 167,320 molecules, and 10,000 of them were randomly chosen for the enriched dataset evaluation analysis. The database was then enriched with 93 known active LDH inhibitors and subjected to the four docking procedures described above.

Virtual screening evaluation: The VS results were assessed through the use of the enrichment factor (EF) and the area under curve (AUC) of the receiver operator characteristic (ROC) curve. The EF measures the enrichment of the method compared with random selection:

EF = [t_p_/(t_p_ + f_n_)](NC_tot_/NC)

where t_p_ is the number of known active ligands retrieved (true positives); f_n_ is the number of known active ligands discarded during the VS filtering (false negatives); NC_tot_ is the total number of compounds of the database; NC is the total number of molecules obtained by the VS protocol [33]. The EF_1%_, EF_5%_ and EF_10%_ indicate the EF values retaining the 1%, 5% and 10% of the whole database. The maximum value that can be reach is 100 (EF_1%_), 20 (EF_5%_) and 10 (EF_10%_); therefore, all of the evaluated EF results were reported as the percentage of these values. The AUC is the area under the ROC curve; an AUC of 0.5 corresponds to a random discrimination between actives and decoys, whereas an AUC very close to 1.0 corresponds to an ideal case, in which all of the known true actives are ranked before all of the decoys.

Molecular dynamics simulations: All simulations were performed using AMBER 11 [34]. The complexes were placed in a rectangular parallelepiped water-box, an explicit solvent model for water (TIP3P) was used; the complexes were solvated with a 10-Å water cap. Chlorine ions were added as counterions to neutralize the system. Prior to MD simulations, two steps of minimization were carried out using the same procedure described above. Particle mesh Ewald electrostatics and periodic boundary conditions were used in the simulation [35]. The MD trajectories were run using the minimized structures as the starting conformations. The time step of the simulations was 2.0 fs with a cutoff of 10 Å for the non-bonded interaction, and SHAKE was employed to keep all bonds involving hydrogen atoms rigid. Constant-volume periodic boundary MD was carried out for 300 ps, during which the temperature was raised from 0 to 300 K. Then, 1.7 ns of constant pressure periodic boundary MD was carried out at 300 K by using the Langevin thermostat to maintain the temperature of our system constant. General Amber force field (GAFF) parameters were assigned to the ligands, while partial charges were calculated using the AM1-BCC method. The MD trajectories were analyzed by using the Ptraj suite of AMBER 11. The ligand’s disposition was monitored, and by using the docking result as a reference pose, all of the ligands that showed an average RMSD greater than 2 Å with respect to the reference disposition were discarded.

### 3.2. Chemistry

#### 3.2.1. General

Commercially available chemicals were purchased from Sigma-Aldrich or Alfa Aesar and used without further purification. Proton (^1^H) and carbon (^13^C) NMR spectra were obtained with a Bruker Avance III 400 MHz spectrometer. Chemical shifts (δ) are reported in parts per million downfield from tetramethylsilane and referenced from solvent references. Chromatographic separations were performed on silica gel columns by flash chromatography (Kieselgel 60, 0.040–0.063 mm; Merck). Reactions were followed by thin-layer chromatography (TLC) on Aldrich aluminum silica gel (F254) sheets that were visualized under a UV lamp. Evaporation was performed *in vacuo* (rotating evaporator). Sodium sulfate was always used as the drying agent. Yields refer to isolated and purified products.

#### 3.2.2. Synthetic Procedures

*Methyl 4-aminobenzoate* (**5**): Commercially available 4-aminobenzoic acid **4** (500 mg, 3.65 mmol) was dissolved in 12.5 mL of methanol, followed by a dropwise addition of sulfuric acid (0.02 mL), and the mixture was refluxed for 48 h. The reaction mixture was cooled to room temperature, and after evaporation of the solvent, the mixture was diluted with water and extracted with EtOAc. The organic phase was dried and concentrated to afford a crude reaction product, which was subjected to flash column chromatography (*n*-hexane/EtOAc 7:3) providing the desired Compound **5** as a white crystalline solid (499 mg, 3.30 mmol, 90% yield). ^1^H-NMR (CDCl_3_): 3.85 (s, 3H), 6.64 (AAʹXXʹ, 2H, *J_AX_* = 8.8 Hz, *J_AA__ʹ/XX__ʹ_* = 2.3 Hz), 7.85 (AAʹXXʹ, 2H, *J_AX_* = 8.8 Hz, *J_AA__ʹ/XX__ʹ_* = 2.3 Hz).

*Methyl 4-((2-hydroxyethyl)amino)benzoate* (**6**): A mixture of methyl ester **5** (200 mg, 1.32 mmol) and 2-iodoethanol (0.07 mL, 0.9 mmol) was heated at 90 °C in a sealed vial for 6 h. The resulting solid was dissolved in ethyl acetate and washed with 2 M aqueous NaOH solution and brine, then dried over Na_2_SO_4_. The solvent was removed under reduced pressure, and the concentrated mixture was purified by flash column chromatography (*n*-hexane/EtOAc 1:1) to obtain the pure amino alcohol **6** as an off-white solid (122 mg, 0.625 mmol, 71% yield). ^1^H-NMR (CDCl_3_): 3.37 (t, 2H, *J* = 5.2 Hz), 3.86 (s, 3H), 3.88 (t, 2H, *J* = 5.2 Hz), 6.63 (AAʹXXʹ, 2H, *J_AX_* = 8.8 Hz, *J_AA__ʹ/XX__ʹ_* = 2.3 Hz), 7.87 (AAʹXXʹ, 2H, *J_AX_* = 8.9 Hz, *J_AA__ʹ/XX__ʹ_* = 2.3 Hz).

*4-((2-Hydroxyethyl)amino)benzoic acid* (**VS1**): Intermediate **6** (50.0 mg, 0.256 mmol) was dissolved in a 1:1 mixture of THF/methanol (2.6 mL) and treated with 0.51 mL of 2 N aqueous solution of LiOH. The reaction was monitored by TLC, and after consumption of the starting material (48 h), the solvents of the mixture were evaporated; then, the residue was diluted with water, treated with 1 N aqueous HCl and extracted with EtOAc. The organic phase was dried and evaporated to afford a crude residue that was purified by flash column chromatography (*n*-hexane/EtOAc 3:7) to obtain the desired Compound **VS1** as a white solid (17.2 mg, 0.0949 mmol, 37% yield). ^1^H-NMR (CD_3_OD): 3.27–3.32 (m, 2H), 3.72 (t, 2H, *J* = 5.8 Hz), 6.62 (AAʹXXʹ, 2H, *J_AX_* = 8.8 Hz, *J_AA__ʹ/XX__ʹ_* = 2.2 Hz), 7.78 (AAʹXXʹ, 2H, *J_AX_* = 8.8 Hz, *J_AA__ʹ/XX__ʹ_* = 2.3 Hz). ^13^C-NMR(CD_3_OD): 46.21, 61.37, 112.21 (2C), 118.49, 132.76 (2C), 154.48, 170.76.

*Methyl 4-(4-methylphenylsulfonamido)benzoate* (**7**): To a solution of aniline **5** (300 mg, 1.98 mmol) in dry CH_2_Cl_2_ (10 mL), pyridine (3.0 mmol, 0.24 mL) and catalytic DMAP (9.3 mg) were added; then, the resulting mixture was cooled to 0 °C. Subsequently, commercially available *p*-toluenesulfonyl chloride (456 mg, 2.39 mmol) dissolved in dry CH_2_Cl_2_ (4 mL) was added dropwise, and the reaction was kept under stirring at RT overnight. The reaction mixture was acidified with 1 N aqueous HCl, extracted with CH_2_Cl_2_, and the organic phase was dried. Evaporation under vacuum of the organic solvent afforded a crude product, which was purified by flash column chromatography (*n*-hexane/Et_2_O 1:1) to yield the sulfonamide derivative **7** as an off-white solid (384 mg, 1.26 mmol, yield 64%). ^1^H-NMR (CDCl_3_): 2.38 (s, 3H), 3.87 (s, 3H), 6.87 (bs, 1H), 7.12 (AAʹXXʹ, 2H, *J_AX_* = 8.8 Hz, *J_AA__ʹ/XX__ʹ_* = 2.2 Hz), 7.23–7.25 (m, 2H), 7.67–7.75 (m, 2H), 7.91 (AAʹXXʹ, 2H, *J_AX_* = 8.8 Hz, *J_AA__ʹ/XX__ʹ_* = 2.2 Hz).

*4-(4-Methylphenylsulfonamido)benzoic* *acid* (**VS2**): Intermediate **7** (100 mg, 0.327 mmol) was dissolved in a 1:1 mixture of THF/methanol (2.6 mL) and treated with 0.40 mL of 2 N aqueous solution of LiOH. The reaction was monitored by TLC; 0.4 mL of 2 N LiOH were added after 24 h, and the mixture was heated at 50 °C. After consumption of the starting material (48 h), the solvents of the mixture were evaporated; then, the residue was diluted with water, treated with 1 N aqueous HCl and extracted with EtOAc. The organic phase was dried and evaporated to afford a crude residue that was purified by flash column chromatography (*n*-hexane/EtOAc 3:7) to obtain the desired compound **VS2** as a pink solid (73.0 mg, 0.251 mmol, 77% yield). ^1^H-NMR (CD_3_OD): 2.36 (s, 3H), 7.17 (AAʹXXʹ, 2H, *J_AX_* = 8.9 Hz, *J_AA__ʹ/XX__ʹ_* = 2.2 Hz), 7.29–7.31 (m, 2H), 7.71 (AAʹXXʹ, 2H, *J_AX_* = 8.4 Hz, *J_AA__ʹ/XX__ʹ_* = 1.8 Hz), 7.85 (AAʹXXʹ, 2H, *J_AX_* = 8.9 Hz, *J_AA__ʹ/XX__ʹ_* = 2.2 Hz). ^13^C-NMR (CD_3_OD): 21.39, 119.78, 128.23 (4C), 130.68 (4C), 131.95, 138.03, 143.53, 145.36.

*5-Phenyl-oxazolidin-2-one* (**9**): To a solution of commercially available 2-amino-l-phenylethanol **8** (500 mg, 3.64 mmol) in CH_2_Cl_2_ (37.4 mL) was added imidazole (124 mg, 1.82 mmol) followed by *N*,*N*-carbonyldiimidazole (620 mg, 3.82 mmol), and the reaction was stirred at room temperature overnight. The mixture was diluted with water and extracted with CH_2_Cl_2_, then with EtOAc. The combined organic phase was dried and evaporated to afford a crude residue, which was purified by flash column chromatography (*n*-hexane/EtOAc 1:1) to obtain Compound **9** as a white solid (411 mg, 2.53 mmol, 70% yield). ^1^H-NMR (CDCl_3_): 3.55 (t, 1H, *J* = 8.2 Hz), 3.99 (t, 1H, *J* = 8.6 Hz), 5.40–5.50 (bs, 1H), 5.63 (t, 1H, *J* = 8.1 Hz), 7.36–7.44 (m, 5H).

*Tert-Butyl 2-(2-oxo-5-phenyloxazolidin-3-yl)acetate* (**10**). To a stirred and cooled solution of 5-phenyl-oxazolidin-2-one **9** (350 mg, 2.16 mmol) in dry DMF (3.5 mL) was added sodium hydride (103 mg of a 60% dispersion in mineral oil, 2.57 mmol). The mixture was stirred at room temperature for 10 minutes; then, *tert*-butyl bromoacetate (505 mg, 2.57 mmol) was added, and stirring was continued for 4 h. The reaction mixture was quenched with ice and H_2_O, treated with 1 N aqueous HCl and extracted with EtOAc. The organic phase was dried and concentrated to obtain a crude residue, which was subjected to flash column chromatography (*n*-hexane/EtOAc 7:3) to afford alkylated derivative **10** as a white solid (413 mg, 1.49 mmol, 69% yield). ^1^H-NMR (CDCl_3_): 1.47 (s, 9H), 3.62 (t, 1H, *J* = 7.9 Hz), 3.96 (ABq, 2H, Δδ_AB_ = 0.05, *J_AB_* = 18.3 Hz), 4.02 (t, 1H, *J* = 8.5 Hz), 5.53 (t, 1H, *J* = 8.2 Hz), 7.35–7.43 (m, 5H).

*2-(2-Oxo-5-phenyloxazolidin-3-yl)acetic acid* (**VS3**): To a solution of intermediate **10** (140 mg, 0.507 mmol) in CH_2_Cl_2_ (3.6 mL) was added dropwise trifluoroacetic acid (0.20 mL), and the reaction was stirred for 26 h, then quenched with ice. After evaporation of the solvent, the residue was carefully neutralized with an aqueous saturated solution of NaHCO_3_, followed by the addition of 1 M aqueous solution of NaOH. The water phase was washed with Et_2_O, then treated with 1 N aqueous HCl and finally extracted with EtOAc. The organic phase was dried, filtered and evaporated to furnish an off-white solid. Formation of a crystalline white precipitate after the addition of hexane resulted in the pure acid **VS3** (151 mg, 0.686 mmol, 99% yield). ^1^H-NMR (CDCl_3_): 3.67 (t, 1H, *J* = 8.0 Hz), 4.06 (t, 1H, *J* = 8.5 Hz), 4.17 (ABq, 2H, Δδ_AB_ = 0.03, *J_AB_* = 18.4 Hz), 4.43 (bs, 1H), 5.59 (t, 1H, *J* = 8.1 Hz), 7.39–7.43 (m, 5H). ^13^C-NMR (CDCl_3_): 45.25, 52.72, 76.10, 126.19 (2C), 129.22 (2C), 129.50, 137.67, 159.31, 172.94.

LDH assays: The LDH inhibition properties of the selected compounds were evaluated against purified human lactate dehydrogenase isoform 5 (Lee Biosolution, Inc., St. Louis, MO, USA). The “forward” direction (pyruvate→lactate) of the lactate dehydrogenase reaction was conducted, and the kinetic parameters were measured by fluorescence (emission wavelength at 460 nm, excitation wavelength at 340 nm) to monitor the amount of consumed NADH. Assays were carried out in wells containing 200 μL of a reagent solution dissolved in 100 mM phosphate buffer (pH = 7.4). For the IC_50_ calculations of the compounds, seven different concentrations (in duplicate for each concentration) of the isolated compounds were used to produce the concentration-response curve. All of the compounds were tested in the presence of 200 μM pyruvate and 40 μM NADH. Any background fluorescence likelihood of the tested samples, or NADH fluorescence quenching, was subtracted. In addition to the sample test wells, maximum and minimum controls were also included in each plate. After 15 min of incubation, the final measurements were carried out by using a Victor X3 Microplates Reader (PerkinElmer^®^). IC_50_ values were produced using GraphPad Prism software. 

Virtual Fragment Scanning: The 3,5 dinitrobenzene portion of Compound **VS8** was virtually replaced with similar fragments by using BROOD software [26]. For the analysis, the default values were used, and the query mask was created, so that the 3,5-dinitrobenzene could be replaced only by fragments possessing an aromatic ring connected with at least three H-bond acceptors in correspondence to the oxygen atoms that showed interactions with R169 and T248. All of the fragments that, when attached to Compound **VS8**, clashed with the protein were eliminated. The filtered compounds were then docked by using the consensus docking procedure described above. All of the compounds possessing a consensus level of 4 and showing H-bonds with R169 and T248 were subjected to MD simulation, and all of the ligands that showed an average RMSD greater than 2 Å with respect to the reference disposition were discarded.

## 4. Conclusions

In this preliminary work, we tested the development of a mixed ensemble/consensus docking platform for identifying new *h*LDH5 inhibitors. The platform was used for filtering the University of Illinois Marvel library of about 10,000 molecules, and the results were further filtered by applying MD simulations. To experimentally verify the reliability of this procedure, ten compounds from the library, which were predicted to be active by the above-mentioned VS study, were tested for their inhibitory potency against *h*LDH5, and two compounds showed IC_50_ values in the high micromolar range. The activity of the newly discovered compounds is quite low and, in another system, would not be considered in the hit range; however, as shown in Table 2, they show an activity that is only about 3–25-fold lower than those of reference LDH inhibitors, and they are characterized by simple molecular structures that can be further functionalized in order to improve their inhibition activity. Taken together, these findings suggest that the optimized techniques herein reported may be suitable for the identification of new *h*LDH5 inhibitors and encourage us to apply this method to other larger databases of compounds and to further refine the VS platform. Furthermore, even if the reported active compounds possess inhibition activities that are lower than those of reference inhibitors, it should be considered that they are small molecules suitable as starting structures for further chemical modifications in order to improve their enzyme inhibition potencies. Therefore, these compounds can be considered as potential hits for the development of new *h*LDH5 inhibitors belonging to novel chemical classes.

## Supplementary Materials

Supplementary materials can be accessed at: http://www.mdpi.com/1420-3049/20/05/8772/s1.
