# Supplementary material for: Development and Validation of a Docking-Based Virtual Screening Platform for the Identification of New Lactate Dehydrogenase Inhibitors
Source: Molecules. 2015 May 15;20(5):8772–90. doi: 10.3390/molecules20058772 (PMC6272605; doi:10.3390/molecules20058772)
Supplement: Supplementary file 1 [file molecules-20-08772-s001.pdf]

Supplementary Materials

Table S1. Active compounds included in the enriched dataset.

#		IC50 (μM)	#		IC50 (μM)
**1**	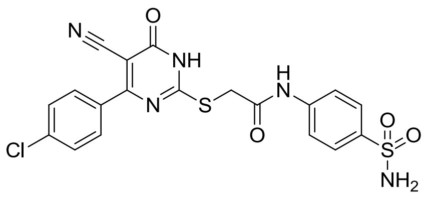	8.8	**2**	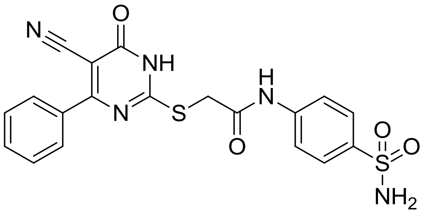	12.1
**3**	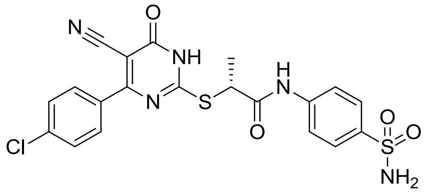	0.48	**4**	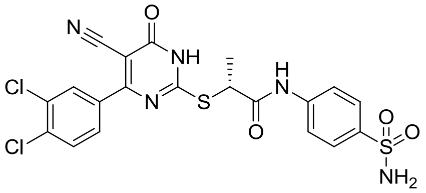	0.75
**5**	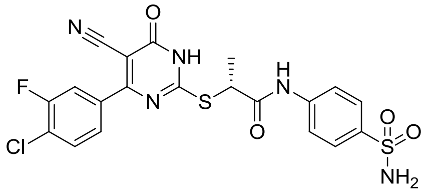	0.71	**6**	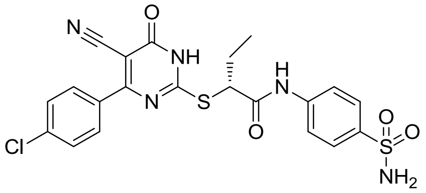	0.65
**7**	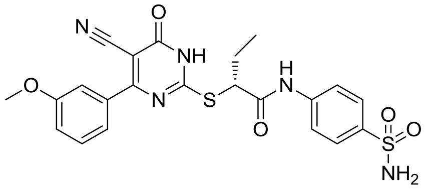	7.4	**8**	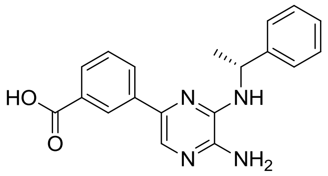	4
**9**	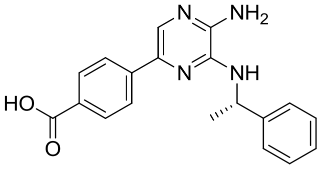	14	**10**	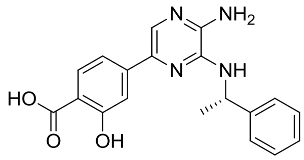	10
**11**	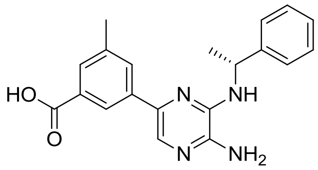	4	**12**	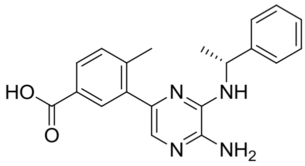	2
**13**	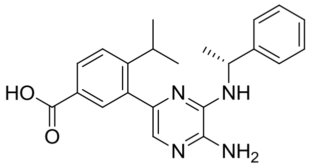	2	**14**	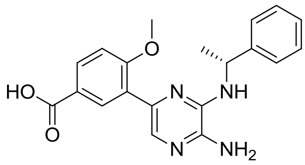	2
**15**	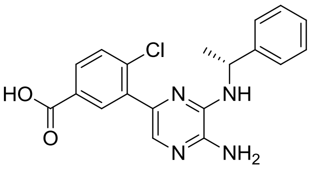	2	**16**	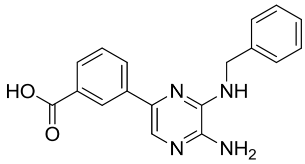	12
**17**	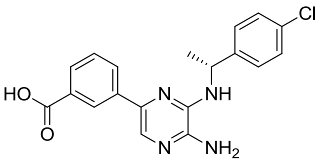	2	**18**	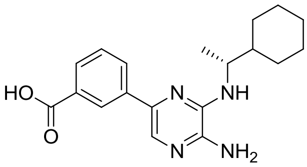	3

Table S1. *Cont.*

#		IC_{50} (μM)	#		IC_{50} (μM)
**19**	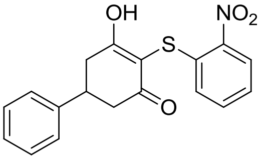	7	**20**	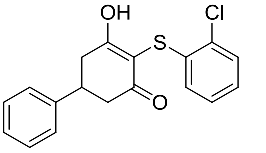	4.6
**21**	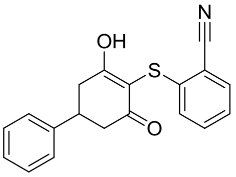	1.6	**22**	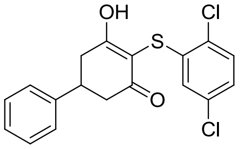	12
**23**	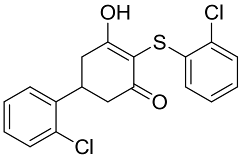	5.6	**24**	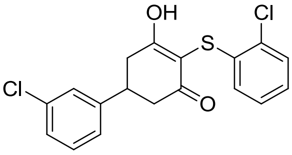	5.6
**25**	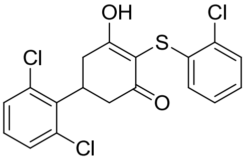	0.87	**26**	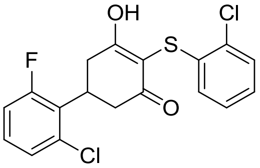	3.7
**27**	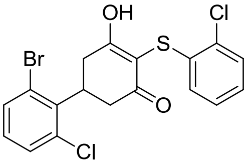	0.58	**28**	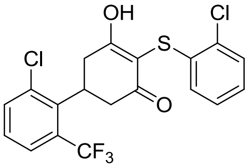	1.0
**29**	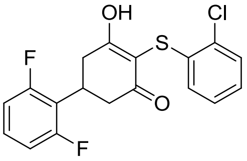	7.4	**30**	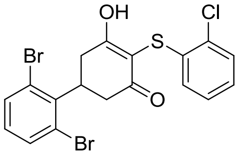	0.27
**31**	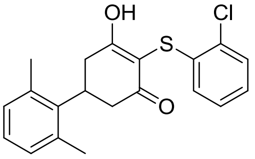	2.3	**32**	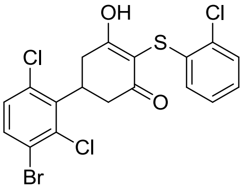	0.5
**33**	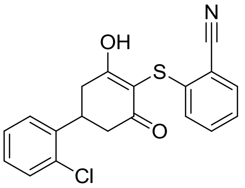	3.1	**34**	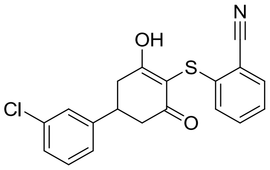	6.8
**35**	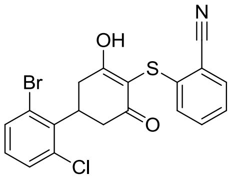	0.45	**36**	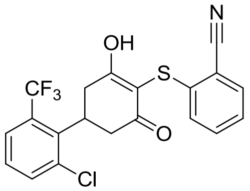	0.64
**37**	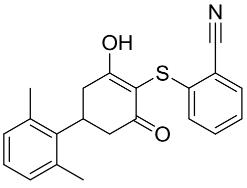	2.1	**38**	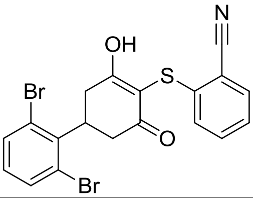	0.18

Table S1. *Cont.*

#		IC_{50} (μM)	#		IC_{50} (μM)
**39**	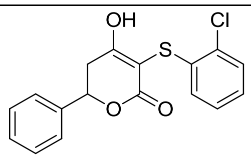	4.0	**40**	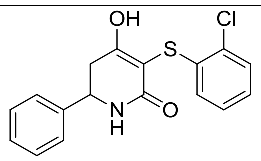	4.0
**41**	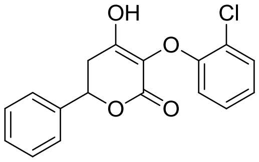	11	**42**	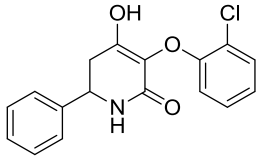	11
**43**	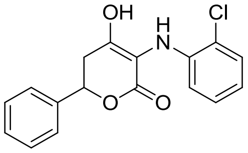	13	**44**	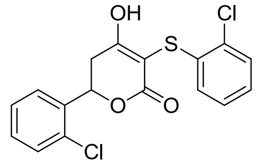	5.5
**45**	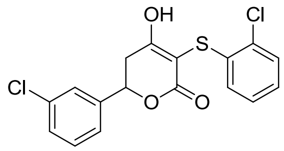	3.9	**46**	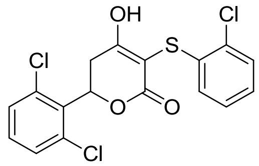	5.0
**47**	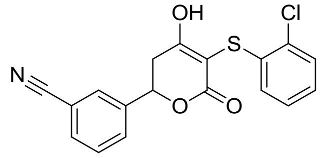	9.5	**48**	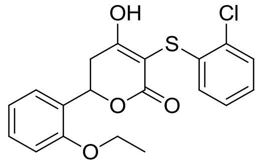	3.8
**49**	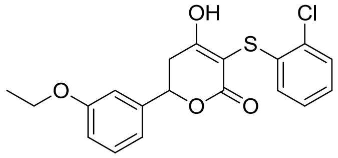	2.2	**50**	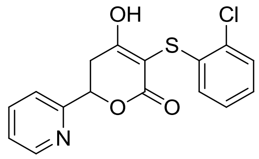	3.5
**51**	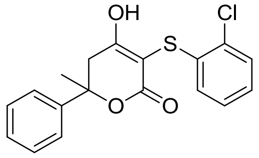	4.1	**52**	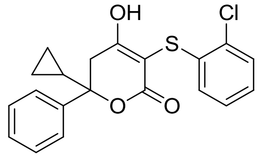	13
**53**	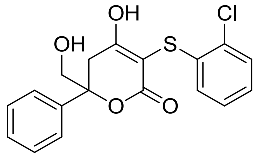	5.6	**54**	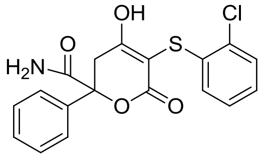	6.6
**55**	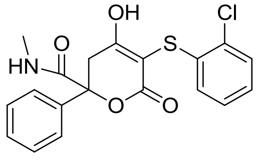	7.4	**56**	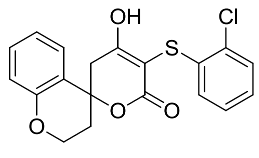	3.3
**57**	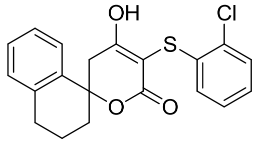	2.1	**58**	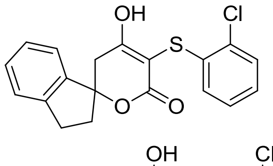	0.36
**59**	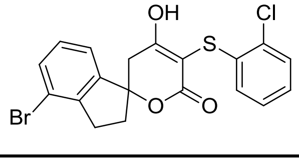	0.35	**60**	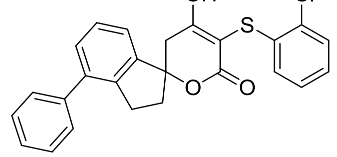	0.03

Table S1. *Cont.*

#		IC_{50} (μM)	#		IC_{50} (μM)
**61**	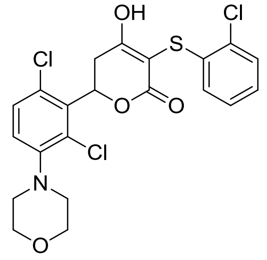	0.24	**62**	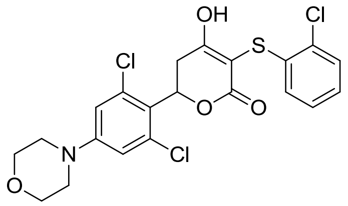	2.6
**63**	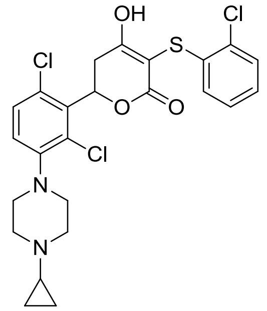	0.23	**64**	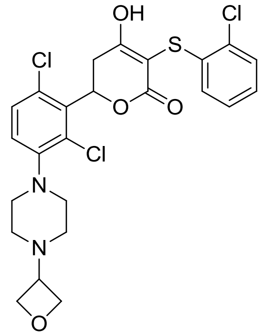	0.37
**65**	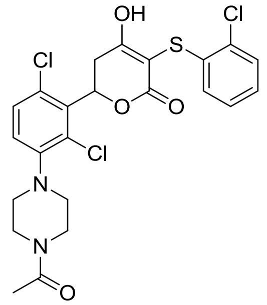	0.10	**66**	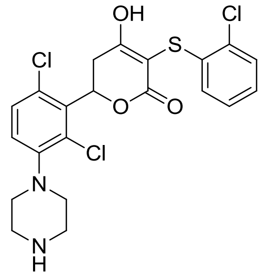	0.17
**67**	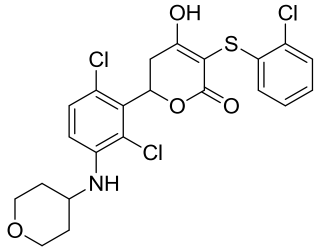	0.06	**68**	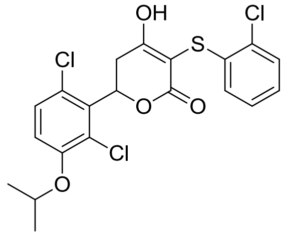	0.09
**69**	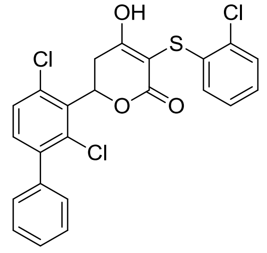	0.10	**70**	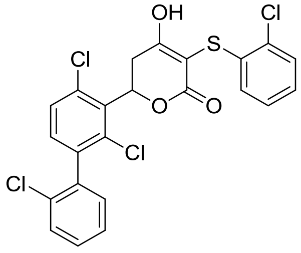	0.06
**71**	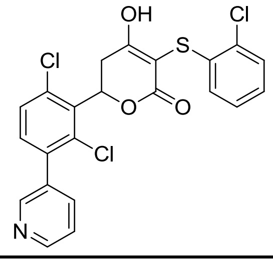	0.108	**72**	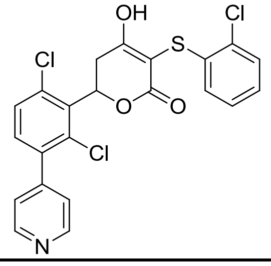	0.085

Table S1. *Cont.*

#		IC50 (μM)	#		IC50 (μM)
**73**	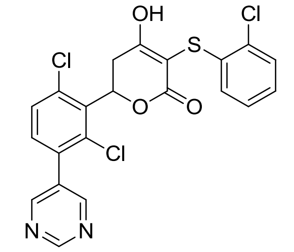	1.1	**74**	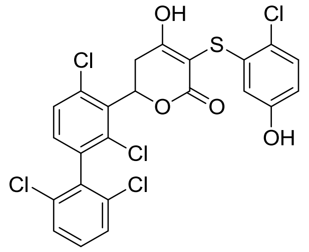	0.12
**75**	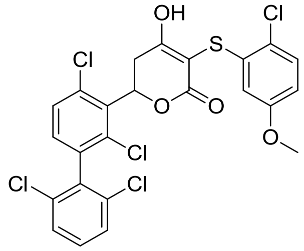	0.37	**76**	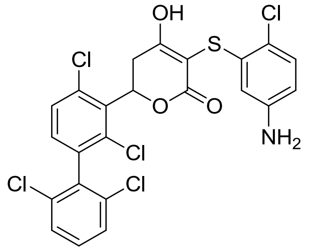	0.64
**77**	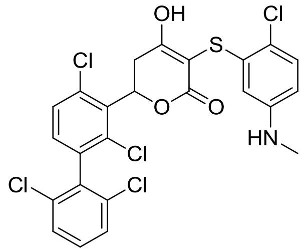	0.17	**78**	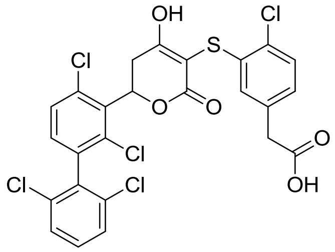	3.5
**79**	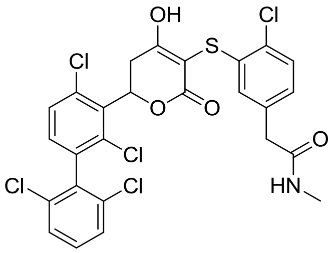	1.8	**80**	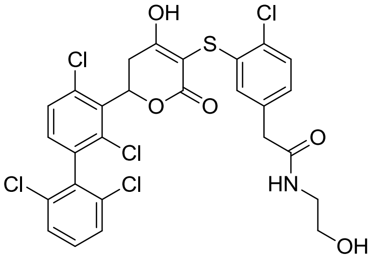	0.3
**81**	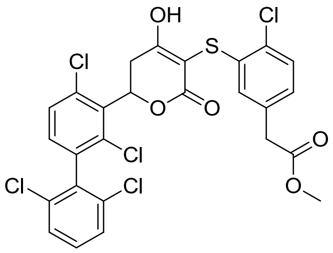	0.04	**82**	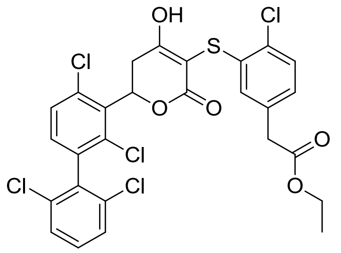	0.13
**83**	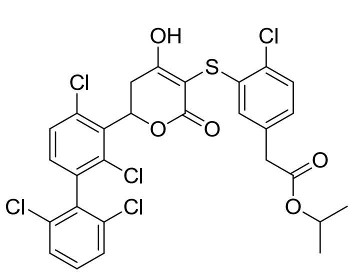	0.12	**84**	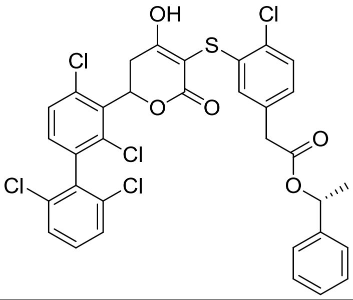	0.006

Table S1. *Cont.*

#		IC_{50} (μM)	#		IC_{50} (μM)
**85**	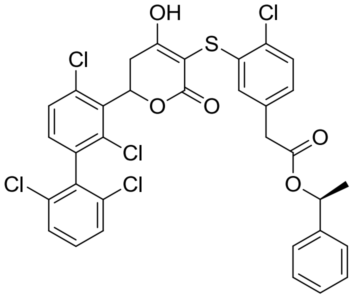	0.09	**86**	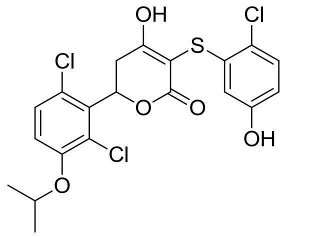	0.03
**87**	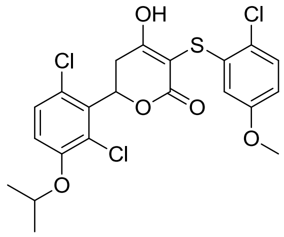	0.025	**88**	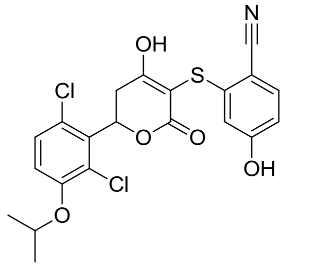	0.015
**89**	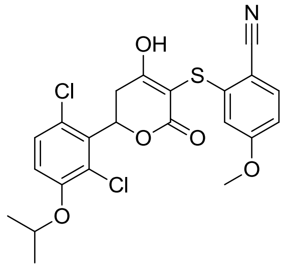	0.46	**90**	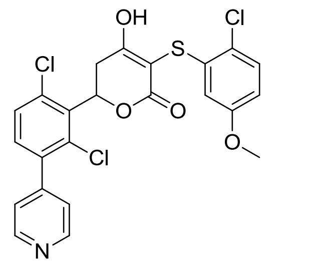	0.035
**91**	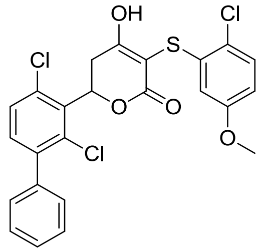	0.030	**92**	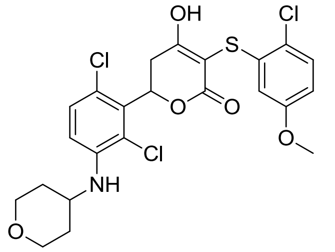	0.49
**93**	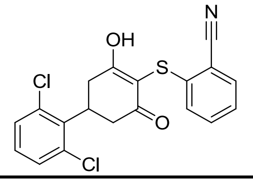	0.87			
